# Draft genome of the brown alga, *Nemacystus decipiens*, Onna-1 strain: Fusion of genes involved in the sulfated fucan biosynthesis pathway

**DOI:** 10.1038/s41598-019-40955-2

**Published:** 2019-03-14

**Authors:** Koki Nishitsuji, Asuka Arimoto, Yoshimi Higa, Munekazu Mekaru, Mayumi Kawamitsu, Noriyuki Satoh, Eiichi Shoguchi

**Affiliations:** 1 Marine Genomics Unit, Okinawa Institute of Science and Technology Graduate University, Onna, Okinawa 904-0495 Japan; 2 Onna Fisheries Cooperative, Onna, Okinawa 904-0414 Japan; 3 DNA Sequencing Section, Okinawa Institute of Science and Technology Graduate University, Onna, Okinawa 904-0495 Japan

## Abstract

The brown alga, *Nemacystus decipiens* (“ito-mozuku” in Japanese), is one of the major edible seaweeds, cultivated principally in Okinawa, Japan. *N*. *decipiens* is also a significant source of fucoidan, which has various physiological activities. To facilitate brown algal studies, we decoded the ~154 Mbp draft genome of *N*. *decipiens* Onna-1 strain. The genome is estimated to contain 15,156 protein-coding genes, ~78% of which are substantiated by corresponding mRNAs. Mitochondrial genes analysis showed a close relationship between *N*. *decipiens* and *Cladosiphon okamuranus*. Comparisons with the *C*. *okamuranus* and *Ectocarpus siliculosus* genomes identified a set of *N*. *decipiens*-specific genes. Gene ontology annotation showed more than half of these are classified as molecular function, enzymatic activity, and/or biological process. Extracellular matrix analysis revealed domains shared among three brown algae. Characterization of genes that encode enzymes involved in the biosynthetic pathway for sulfated fucan showed two sets of genes fused in the genome. One is a fusion of l-fucokinase and GDP-fucose pyrophosphorylase genes, a feature shared with *C*. *okamuranus*. Another fusion is between an ST-domain-containing gene and an alpha/beta hydrolase gene. Although the function of fused genes should be examined in future, these results suggest that *N*. *decipiens* is another promising source of fucoidan.

## Introduction

Brown algae comprise many types of seaweeds in oceans and serve important functions in marine ecosystems^1^. Taxonomically, brown algae belong to the very large Class Phaeophyceae, in the division Heterokontophyta. They are eukaryotes, distinguished by having chloroplasts surrounded by four membranes^2^. This suggests that they arose from a symbiotic relationship between a basal eukaryote and another eukaryotic organism with chloroplasts. Here we examine a brown alga, *Nemacystus decipiens*. The phylogenetics of *N*. *decipiens* and a closely related species, *Cladosiphon okamuranus*, are a matter of some debate. According to Silberfeld *et al*.^3^, both *N*. *decipiens* and *C*. *okamuranus* are classified as members of the family Chordariaceae of the order Ectocarpales. On the other hand, Migita and Yotsuji^4^ and Yoshida *et al*.^5^ classified *N*. *decipiens* as a member of the family Spermatochnaceae of the order Chordariales, and *C*. *okamuranus* is as a member of the family Chordariaceae within the same order. In this report, we adopt the latter classification.

**Figure 1:**
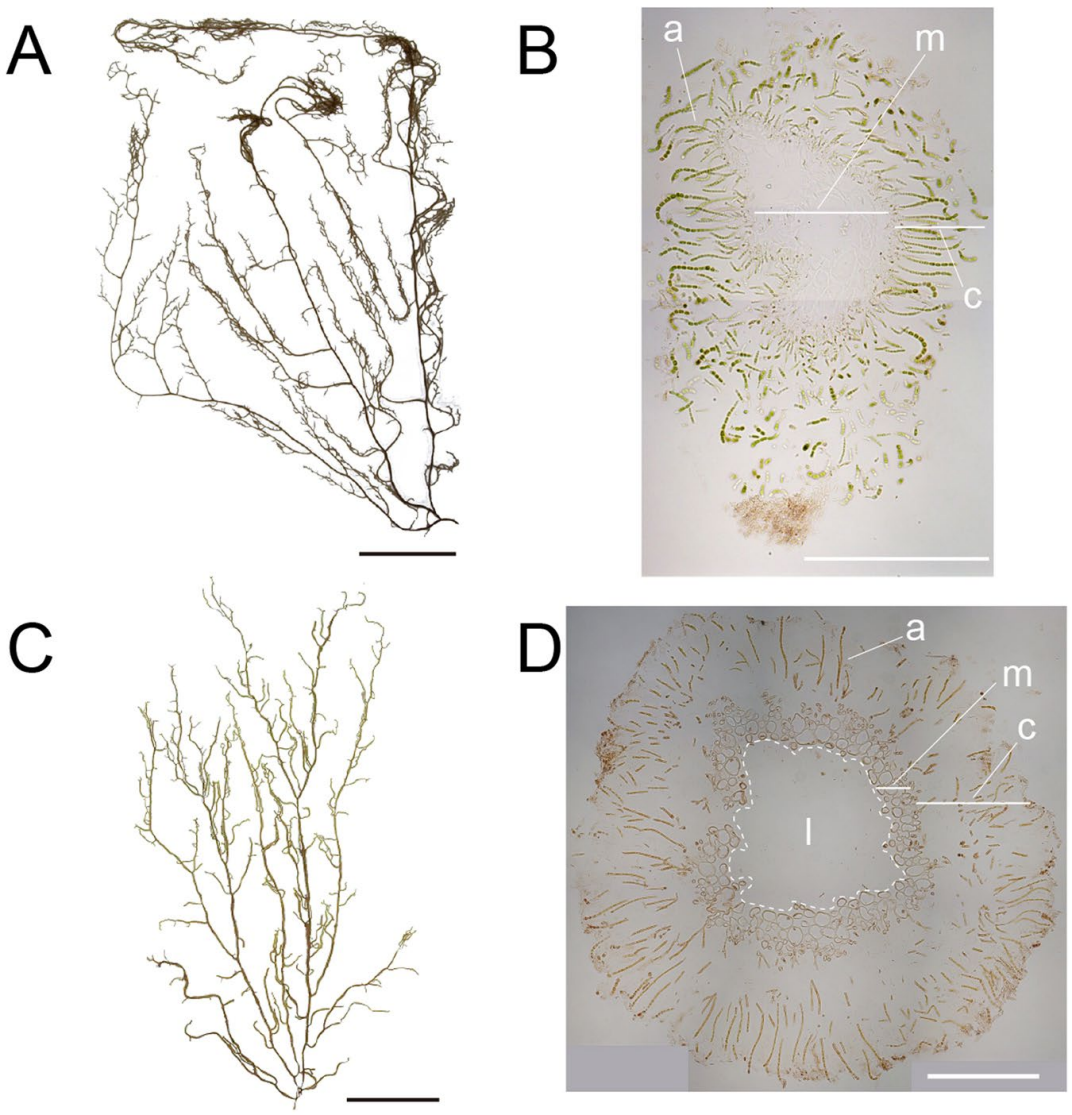
*Nemacystus decipiens* (**A,** ito-mozuku) and *Cladosiphon okamuranus* (**C,** Okinawa mozuku) have fibrous sporophytes, less than 1 mm and 1~2 mm in diameter, respectively (**B**,**D**). Those sporophytes have cortexes that contain assimilatory filaments surrounding the medullas. Sporophytes of *C*. *okamuranus* have lumens. a, assimilatory filament; c, cortex; l, lumen; m, medulla; Scale bar, 10 cm (**A**,**C**) and 500 µm (**B**,**D**). The photograph of *C*. *okamuranus* sporophyte was provided by Mr. Kenji Iwai under a CC BY open access license.

Brown algae provide food resources^6^. Major cultured seaweeds in Japan include *Laminaria*, *Saccharina*, *Undaria*, *Cladosiphon*, and *Nemacystus*. In Okinawa, *C*. *okamuranus* and *N*. *decipiens* represent major food products. *N*. *decipiens* (“ito-mozuku” in Japanese) and *C*. *okamuranus* (“Okinawa mozuku” in Japanese) are morphologically similar (Fig. 1A,C). Both have frond-like sporophytes, and the diameter of main axes is less than 1 mm in the former and 1~2 mm in the latter. Sporophytes are composed of cortexes in the outer layer and the medullas in the inner layer, the former of which contains assimilatory filaments. The two algae are distinguishable by a lumen-like space found only in *C*. *okamuranus* sporophytes (Fig. 1B,D). *N*. *decipiens* and *C*. *okamuranus* have been cultivated in Okinawa for more than 25 and 35 years, respectively (Supplementary Fig. S1A). Cultivation has established 19 strains of *N*. *decipiens* and 5 of *C*. *okamuranus*. It is reported that approximately 800 tons of *N*. *decipiens* were produced in fiscal year 2017, versus ~17,000 tons of *C*. *okamuranus*.

In addition, brown algae produce carotenoids, including fucoxanthin, which is essential for photosynthesis. Brown algae also produce fucoidan^7^, one of the sulfated polysaccharides found in the cell-wall matrix of brown algae. It has anticoagulant, antithrombin, and antitumor activities^8^. Brown algae also known synthesize alginates^9,10^. Therefore, they are a source of important biomaterials in the fisheries industry.

Due to global environmental changes, including temperature increase, acidification, and pollution, brown algal aquaculture is facing critical conditions^11^. Continuous efforts to maintain and improve culture methods are required, and genomic information is essential for this. So far, the genomes of *Ectocarpus siliculosus*^12^ (Order Ectocarpales), *Saccharina japonica*^13^ (Order Laminariales), and *Cladosiphon okamuranus*^14^ (Order Chordariales) have been decoded. In a previous study, we decoded the ~130-Mbp genome of *C*. *okamuranus*, which is a good fucoidan producer (250 milligram per gram dry weight)^7,15^. We identified and characterized genes for enzymes involved in the sulfated fucan biosynthetic pathway^14^. l-fucokinase phosphatizes l-fucose to fucose-1-phosphate and GDP-fucose pyrophosphorylase catalyzes fucose-1-phosphate to GDP-fucose. We isolated mRNA indicating that in *C*. *okamuranus*, these two genes have fused, which may be advantageous for producing fucoidan. *N*. *decipiens* has also been recognized as a good source of fucoidan (250 milligram per gram dry weight)^7,15^. In this study, we decoded a draft genome of *Nemacystus decipiens* and compared it to other brown algal genomes.

## Results

### Genome sequencing and assembly

Details of sequencing and genome assembly are presented in Supplementary Table S1. The Illumina MiSeq platform (average library size, 700 base-pair (bp)) generated a total of 47.1 giga-base-pair (Gbp) of paired-end sequence data (average read length = 309 bp). The HiSeq 4000 platform generated a total of 33.0 Gbp of mate-pair sequences; 4.3 Gbp for 2 kb, 4.4 Gbp for 3 kb, 4.3 Gbp for 4 kb, 4.4 Gbp for 5 kb, 4.4 Gbp for 6 kb, 4.2 Gbp for 7 kb, 3.0 Gbp for 9 kb, 2.2 Gbp for 11 kb, and 1.9 Gbp for a 13 kb library (average read length 151 bp). A total of 80.1 Gbp of sequences data was obtained (Supplementary Table S1).

The genome size of *N*. *decipiens* was estimated by counting K-mer frequencies of raw reads (K-mer = 32). In Supplementary Fig. S2A, the peak appeared at around ~95. The calculated genome size was ~190 Mbp. A total read of 80.1 Gbp would correspond to approximately 420-fold sequencing coverage of the estimated genome.

**Table 1:** Comparison of draft genome assemblies of three brown algae, *Nemacystus decipiens* (Order Spermatochnaceae), *Cladosiphon okamuranus* (Order Chordariales), and *Ectocarpus siliculosus* (Order Ectocarpales).

	Species		
	*Nemacystus decipiens* ^a^	*Cladosiphon okamuranus* ^b^	*Ectocarpus siliculosus* ^c^
Total length (Mbp)	154	130	197
Number of scaffolds	685	541	30
N50 Scaffold size (kbp)	1,863	418	6,528
Number of contigs	411,597	31,858	—
N50 contig size (bp)	6,265	21,705	—
Number of genes	15,156	12,999	17,418
Average gene length (bp)	7,902	7,949	7,542
Average number of introns per gene	10.24	9.14	6.96
Average intron length (bp)	588	530	740
GC Contents (%)	56	54	54
Repeated sequences (%)	8.8	11.2	22.7
CEGMA Completeness (%)	84.3	83.1	72.6
CEGMA Partial (%)	93.6	88.3	87.5

^a^The present study.

^b^Nishitsuji *et al*. *DNA Res*. **23**, 561–570 (2016).

^c^Cormier *et al*. *New Phytol*. **214**, 219–232 (2017).

Illumina paired-end reads were assembled *de novo* using Platanus. The assembled genome contained 411,597 contigs with an N50 size of 6,265 bp (Table 1). The longest contig was 135,338 bp, and approximately 47% of sequences were covered with contigs over 2 kb in length. Subsequent scaffolding of 411,597 Platanus output was performed with SSPACE, using Illumina mate-pair sequence information (Supplementary Table S1). Gaps inside the scaffolds were closed with GapCloser. Contaminating bacterial and microbial scaffolds identified using Maxbin and RNAmmer were deleted. Final assembly of the *N*. *decipiens* genome was 685 scaffolds with an N50 size of 1.863 Mbp. Total length of scaffolds reached 154 Mbp (Table 1).

CEGMA analysis indicated 93.6% sequences for partial yields and 84.3% sequences for complete yields (Table 1). For comparison, CEGMA partial and complete values for genome sequences of *C*. *okamuranus* and *E*. *siliculosus* are 88.3% and 87.5%, and 83.1% and 72.6% (Table 1), respectively. This suggests that the assembled genome of *N*. *decipiens* has the higher quality of the three brown algal genomes.

### GC content

The GC content of the *N*. *decipiens* genome was calculated as ~56% (Supplementary Fig. S2B; Table 1), versus 54% for both *C*. *okamuranus* and *E*. *siliculosus* (Table 1).

### RNA-seq, assembling, and mapping

Transcriptomic data are essential to analyze composition and expression of genes. RNA extracted from protonemas (Supplementary Fig. S1B) was sequenced using the HiSeq. 4000 platform (average library size was 260 nucleotides (nts), and read length 151 nts) (Supplementary Table S1). A total of 28.5 giga nts were generated. Transcripts assembled with the Velvet/Oases yielded 204,065 contigs (a total of 345 mega nts) with an N50 size of 3,313 nts. 152,212 (74.6%) assembled transcripts were aligned to the assembled genome (with default settings) with blat software. These data were used to produce gene models and annotations.

### Gene modeling

Assembled RNA sequences and putative protein coding loci found with blastx were incorporated as AUGUSTUS “hints.” The number of gene models was 15,156 (Table 1). This is larger than the 12,999 predicted genes of *C*. *okamuranus* (on 541 scaffolds, version 2: http://marinegenomics.oist.jp/algae/viewer/download?project_id=67), and fewer than the 17,418 predicted genes of *E*. *siliculosus* (version 2: https://bioinformatics.psb.ugent.be/gdb/ectocarpusV2/)^16^. The average length of *N*. *decipiens* genes was 7,902 bp and that of exons (coding sequences) was 2,710 bp.

The *C*. *okamuranus* and *E*. *siliculosus* genomes are intron-rich^12,14^; average numbers of introns per gene are 9.14 and 6.96, and average intron lengths are 530 bp and 740 bp, respectively (Table 1). This feature was more prominent in the *N*. *decipiens* genome. The average number of introns per gene was 10.24, and the average length of an intron was 588 bp (Table 1). Land plants and non-brown algae have lower average numbers of introns per gene; 5.43 in *Arabidopsis thaliana*, 4.39 in *Oryza sativa* ssp. *japonica*, 3.89 in *Hordeum vulgare*, 4.35 in *Zea mays*, 5.34 in *Physcomitrella patens*, 5.69 in *Marchantia polymorpha*, 6.63 in *Klebsormidium nitens*, 3.82 in *Chara braunii* and 8.07 in *Chlamydomonas reinhardtii*, respectively^17^. This feature of brown algal genes should be examined in future.

### Transposable elements and other repetitive components

We examined the proportion of transposable elements and repetitive elements in the assembled *N*. *decipiens* genome. DNA transposons and retrotransposons accounted for 0.2098% and 2.0143% of the *N*. *decipiens* genome, respectively (Supplementary Table S2). DNA transposons included EnSpm (0.0440% of assembled sequences), Helitron (0.0186%), hAT (0.0157%), and Polinton (0.0110%). Retrotransposons included LTR (long terminal repeat) retrotransposons such as Gypsy (0.8189%), Copia (0.4700%), and Bel_Pao (0.0681%), and the non-LTR retrotransposon CR1 (0.0016%). Percentages for LINE (long interspersed nuclear elements) are 0.0733% for Jockey, 0.0458% for Tx1 and 0.0072% for L1, and that for SINE (short interspersed nuclear elements) is 0.0024%. Repetitive sequences, including unclassified repeats comprised 8.8% of the *N*. *decipiens* genome (Supplementary Table S2). This is less than the two other brown algae, i.e.,11.2% for *C*. *okamuranus* and 22.7% for *E*. *siliculosus*, respectively (Table 1). An interesting question for future studies is how the variation in quality and quantity of repetitive sequences affects the composition of brown algal genomes.

### Genome browser

A genome browser has been established at: http://marinegenomics.oist.jp/ito_mozuku_v1/viewer/info?project_id=68. Gene annotations from domain searches and Blast2GO^18^ are provided on the site.

### Phylogenetic position of *Nemacystus decipiens*

**Figure 2:**
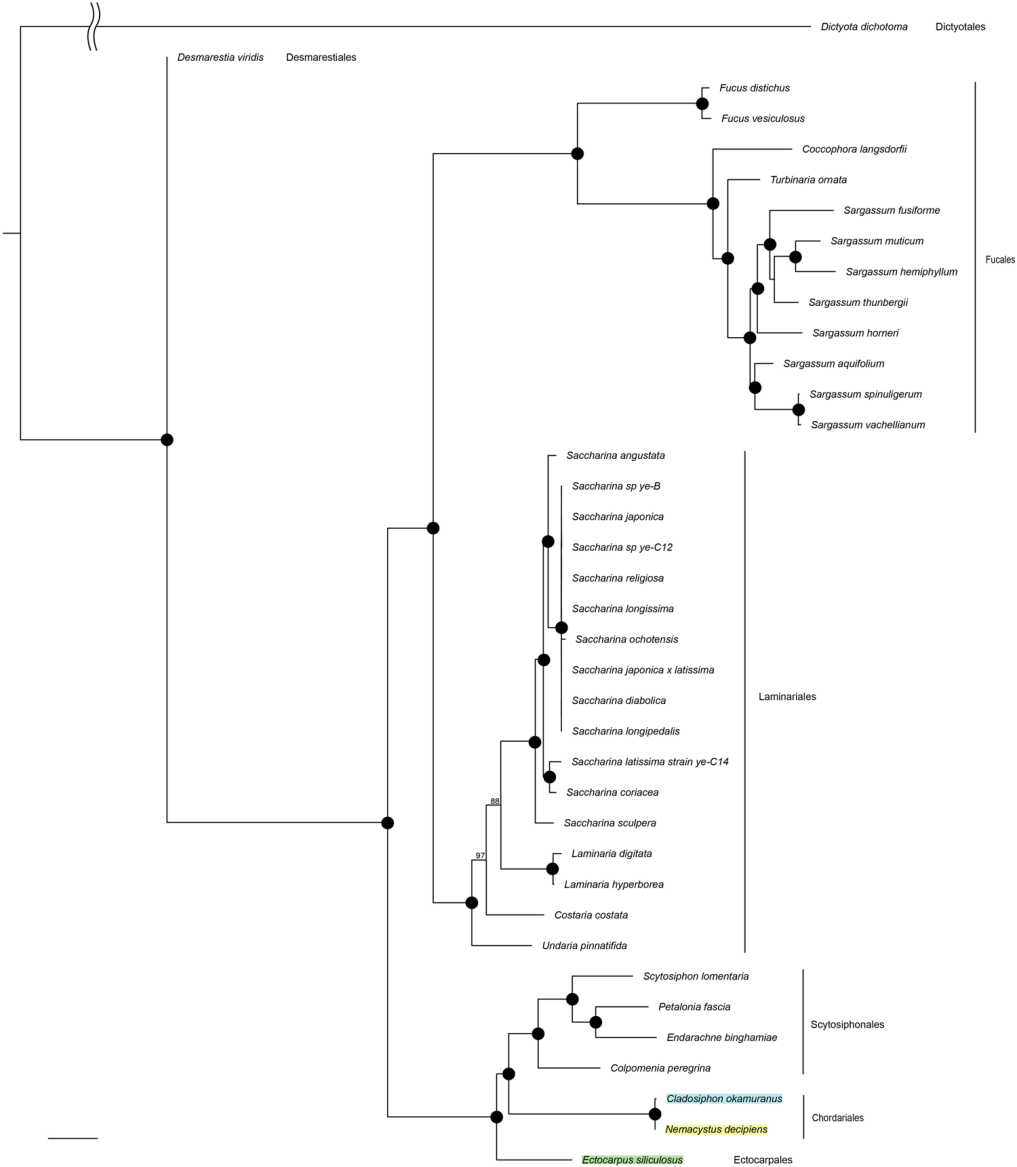
Phylogenetic tree of 38 brown algae based on a comparison of 32 mitochondrial protein-coding gene sequences. *Nemacystus decipiens* forms a clade with *Cladosiphon okamuranus*, that may correspond to the order Chordariales. On the other hand, *Ectocarpus siliculosus* forms another clade in the order Ectocarpales. Full tree was shown in Fig. S3. Black dots represent 100% bootstraps. Scale bar, 0.1 substitutions/site.

Based on morphological and molecular criteria, *N*. *decipiens* was classified as belonging to the family Spermatochnaceae of the order Chordariales^4,5^. On the other hand, *C*. *okamuranus* has been classified into the family Chordariaceae of the same order. Another brown alga, *E*. *siliculosus*, belongs to the order Ectocarpales. To examine phylogenetic relationship of the three algae, we carried out molecular phylogenetic analysis based on a comparison of nucleotide sequences of 32 protein-coding genes in mitochondria genomes of 38 brown algae. As shown in Fig. 2 and Supplementary Fig. S3, N. *decipiens* and *C*. *okamuranus* form a clade corresponding to the order Chordariales while *Scytosiphon lomentaria* and three other species form a clade corresponding to the order Scytosiphonales, and *E*. *siliculosus* belongs to an independent clade of the order Ectocarpales (Fig. 2 and Supplementary Fig. S3). This indicates *N*. *decipiens* and *C*. *okamuranus* share a more recent common ancestor.

### Transcription factor genes

We searched for genes that encode transcription factors (TFs) in the *N*. *decipiens* genome using hmmer3 and the Pfam database (e-value cutoff <e^−5^), and compared them with those in the *C*. *okamuranus*^14^ and *E*. *siliculosus*^16^ genomes (Supplementary Table S3). The domains include HSF, Myb, bZIP, Zinc Finger, bHLH, CCAAT-binding, Homeobox, AP2-EREBP, Nin-like, TAF, E2F-DP, CBF/NF-Y/archaeal, and Sigma-70 r2/r3/r4 (Supplementary Table S3). It appears that the *N*. *decipiens* genome contains 299 transcription factor genes (Supplementary Table S3), versus 257 in the *C*. *okamuranus* genome (version 2) and 274 in the *E*. *siliculosus* genome (version 2), suggesting a small expansion of the TF family in *N*. *decipiens*. The most abundant TFs occurred in the Myb family, with 79, 74, and 70 genes detected in *N*. *decipiens*, *C*. *okamuranus*, and *E*. *siliculosus* genome, respectively. Others that were plentiful in the *N*. *decipiens* genome were CBF/NF-Y/archaeal (42), bZIP (36), Sigma-70 r2/r3/r4 (32), Zinc Finger C2H2-type (26), Zinc Finger CCCH-type (22), and HSF (22). The *N*. *decipiens* genome contains four genes with bHLH domains, three with homeobox domains, and ten with TAF domains, respectively.

### Comparison of orthologous gene groups

**Figure 3:**
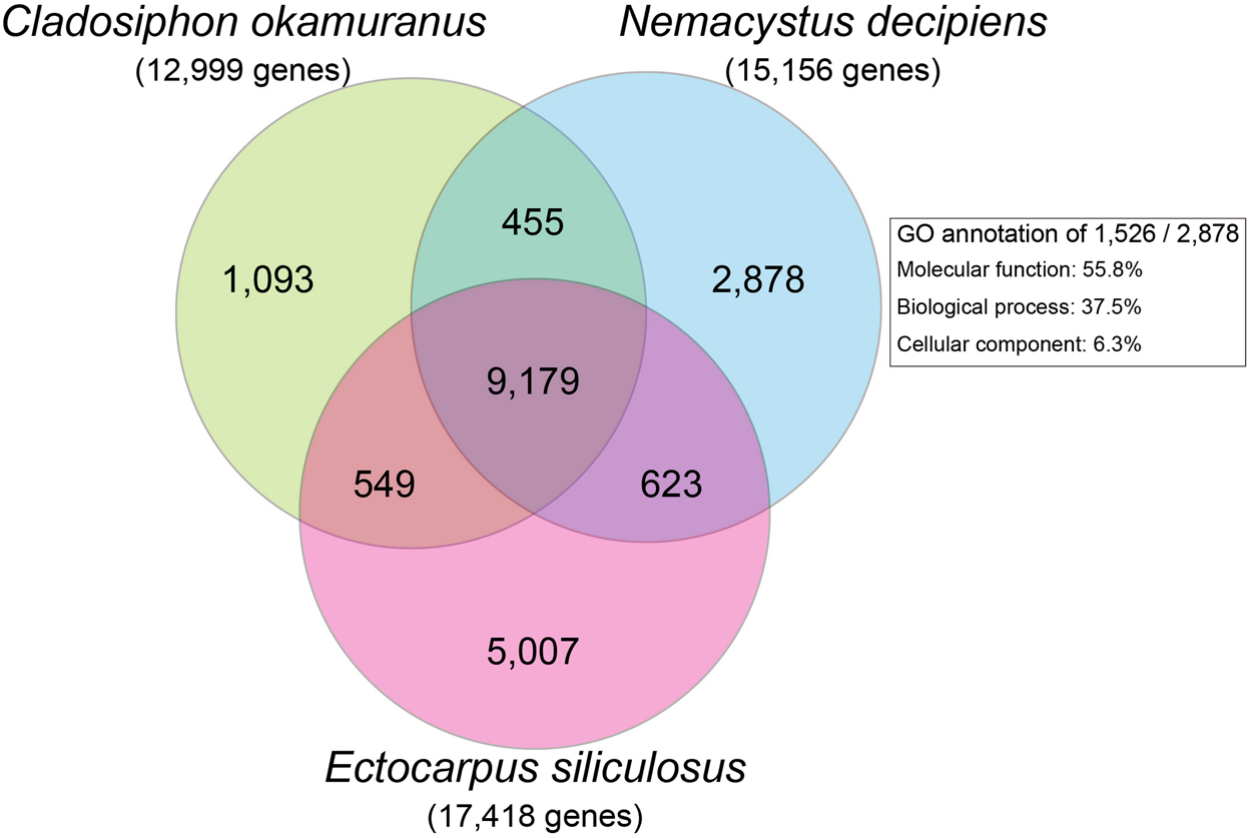
Numbers of orthologous gene groups among the three brown algae, *Nemacystus decipiens*, *Cladosiphon okamuranus*, and *Ectocarpus siliculosus*.

The *Nemacystus* genome contains 15,156 gene models, which is comparable to the genomes of *Cladosiphon* (12,999) and *Ectocarpus* (17,418)^14,16^. A total of 9,179 orthologous gene groups were conserved among the three algae (Fig. 3). In addition, 455 orthologous groups were shared by *N*. *decipiens* and *C*. *okamuranus*, 549 by *C*. *okamuranus* and *E*. *siliculosus*, and 623 by *N*. *decipiens and E*. *siliculosus*. 2,878, 1,093, and 5,007 groups were found to be unique in genomes of *N*. *decipiens*, *C*. *okamuranus*, and *E*. *siliculosus*, respectively. 1,526 of the 2,878 unique groups in the *N*. *decipiens* genome could be GO-annotated (Supplementary Table S4). Among these, 55.8% were categorized as “molecular function” 37.5% as “biological process,” and 6.3% as “cellular component.” This indicates that many genes unique to *N*. *decipiens* may not be involved in cellular structure or composition, but in physiological processes such as alanine dehydrogenase and xanthine phosphoribosyl transferase activity. In fact, many of these genes encoded enzymes involved in polysaccharide biosynthetic processes (Supplementary Table S5). Furthermore, 617 of 1,352 non-GO-annotated gene groups were not found in the non-redundant protein sequence database at NCBI, and 200 of the 617 genes were annotated (Supplementary Table S6).

### Extracellular matrix genes

**Figure 4:**
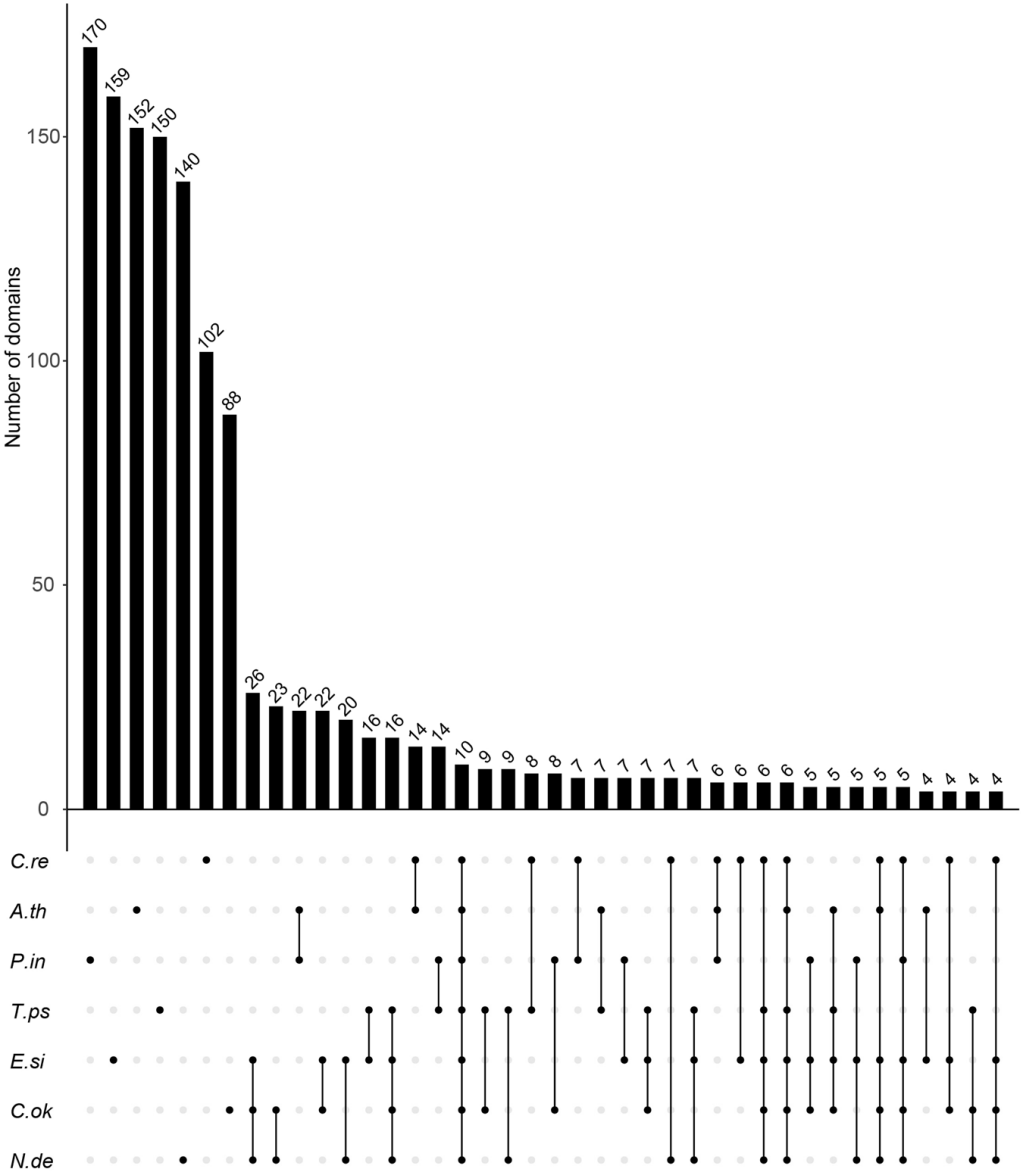
Upset plot representing domains associated with extracellular matrix in seven organisms. The 40 most abundant domains in the extracellular matrix are shown. *N*.*de* indicates *Nemacystus decipiens*; *C*.*ok*, *Cladosiphon okamuranus*; *E*.*si*, *Ectocarpus siliculosus*; *T*.*ps*, *Thalassiosira pseudonana*; *P*.*in*, *Phytophthora infestans*; *A*.*th*, *Arabidopsis thaliana*; and *C*.*re*, *Chlamydomonas reinhardtii*. A set of 26 and additional set of 23 domains were shared among the three brown algae and among Chordariales, respectively. On the other hand, 140, 88, and 159 domains were unique in the genomes of *N*. *decipiens*, *C*. *okamuranus*, and *E*. *siliculosus*, respectively. The 10 domains were common in the seven organisms.

The extracellular matrix (ECM) is composed of collagens, elastin, and proteoglycans, elements of which are polysaccharides and glycoproteins^19–21^. It regulates morphogenesis, cell differentiations, evolution of multicellularity, and cell-to-cell communication, and responses to stimuli from the environment^19–21^. In order to examine brown algae-unique and Chordariales (*N*. *decipiens* and *C*. *okamuranus*)-unique ECM components, we searched genes for those possibly associated with the ECM in genomes of the three brown algae, a diatom (*Thalassiosira pseudonana*), an oocyte (*Phytophthora infestans*), a green alga (*Chlamydomonas reinhardtii*), and a land plant (*Arabidopsis thaliana*), as described in the Materials and Methods. 676, 649, 901, 644, 1,116, 699, and 1,116 genes were defined as putative ECM genes in *N*. *decipiens*, *C*. *okamuranus*, *E*. *siliculosus*, *T*. *pseudonana*, *P*. *infestans*, *C*. *reinhardtii*, and *A*. *thaliana* genomes, respectively (Supplementary Tables S7 and S8). These genes were annotated with the Pfam database and the number of annotated domains was counted. As a result, 140, 88, and 159 unique domains were found in *N*. *decipiens*, *C*. *okamuranus*, and *E*. *siliculosus*, respectively (Fig. 4). 26 domains were shared among the three brown algae, and additional 23 domains were conserved in the order Chordariales (Fig. 4). One GlcNAc gene (PF11397.6) that was also annotated as glycosyl transferase family 60 was found in each of the three genomes. On the other hand, three and two glycosyl transferase family 2 genes (PF13704.4) was found only in *N*. *decipiens* and *C*. *okamuranus* genomes, respectively (Supplementary Tables S8). Glycosyl transferase is necessary for polysaccharide biosynthesis^22^. Although function of the gene has not been analyzed yet, the results suggest that *N*. *decipiens* and *C*. *okamuranus* evolved recently from a common ancestor that had acquired the glycosyl transferase family 2 gene, and that the GlcNAc gene may play an important role in polysaccharide biosynthesis in the brown algae.

### Genes associated with fucoidan biosynthesis

**Figure 5:**
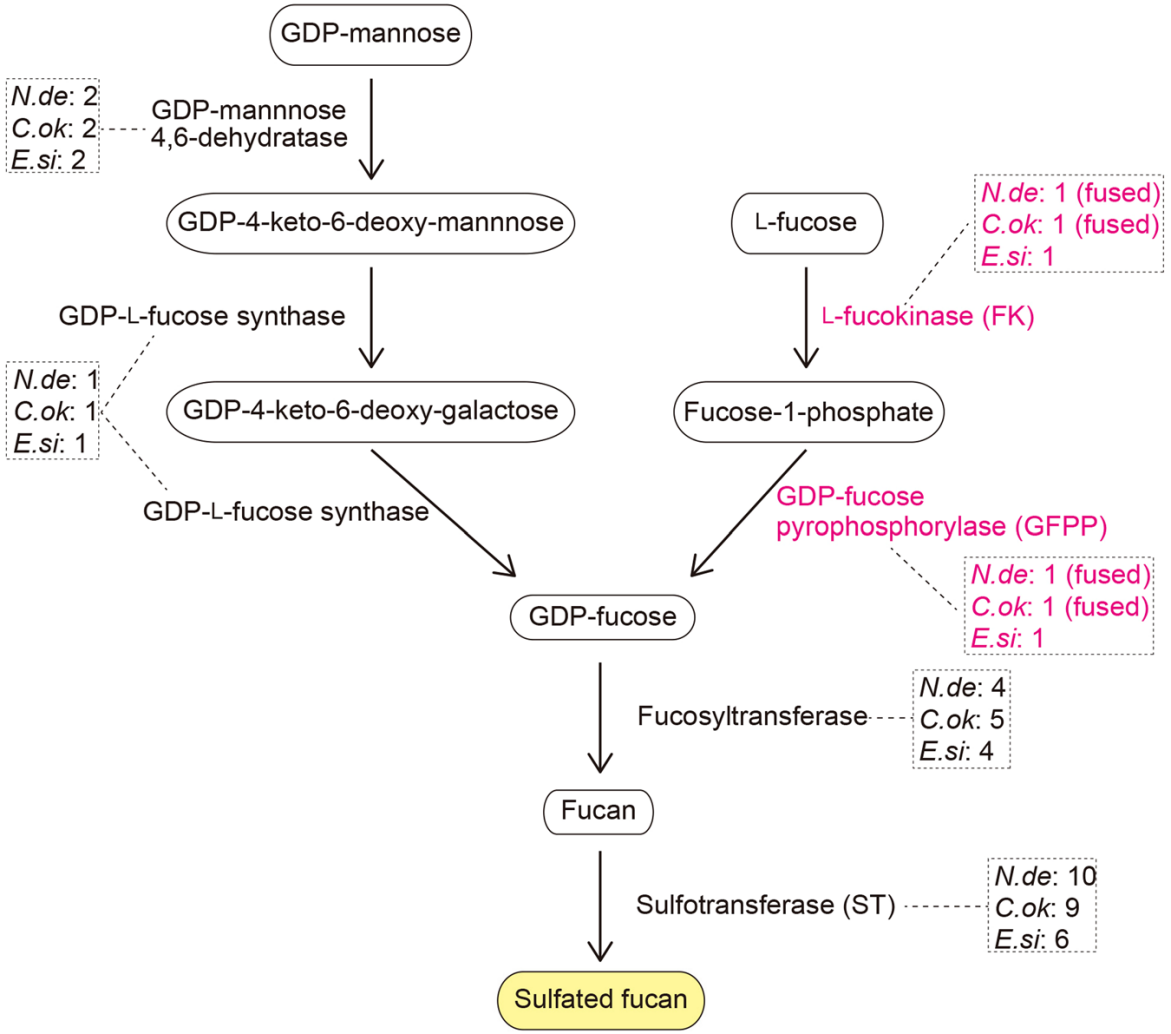
Identification of genes for enzymes in the biosynthetic pathway of sulfated fucan in three brown algae. Gene numbers in each genome are also shown. *N*.*de*: *Nemacystus decipiens*. *C*.*ok*: *Cladosiphon okamuranus*. *E*.*si*: *Ectocarpus siliculosus*.

Fucoidans are a family of sulfated homo- and hetero-polysaccharides of brown algae that contain l-fucose residues. The family comprises a broad spectrum of polysaccharides, from compounds with high uronic acid content and low fucose and sulfate content to almost pure α-l-fucan with fucose as the dominant monosaccharide. Genes encoding key enzymes for polysaccharide metabolism in brown algae were first predicted from the *E*. *siliculosus* genome^10^. Six enzymes are involved in this pathway (Fig. 5). GDP (guanosine diphosphate)-mannose and l-fucose are original sources of GDP-fucose, which are transformed to sulfated fucan via fucan (Fig. 5).

With a Blast search, our previous analyses indicated that genes encoding these key enzymes are conserved between *C*. *okamuranus* and *E*. *siliculosus*, although those for downstream enzymes are likely expanded independently in each lineage (Fig. 5)^14^. Specifically, the *C*. *okamuranus* and *E*. *siliculosus* genomes each contain two genes for GDP-mannose 4,6-dehydratase, and one gene for GDP-l-fucose synthase (Fig. 5). Both genomes hold one gene for l-fucokinase (FK) and one gene for GDP-fucose pyrophosphorylase. We found that the *N*. *decipiens* genome contained the same number of genes for the four enzymes (Fig. 5). The number of fucosyltransferases and sulfotransferases is variable among the three brown algae (Fig. 5). The *N*. *decipiens*, *C*. *okamuranus*, and *E*. *siliculosus* genomes contain four, five, and four genes for fucosyltransferase, and ten, nine, and six genes for sulfotransferase, respectively (Fig. 5; details of this information are in Supplementary Tables S9).

**Figure 6:**
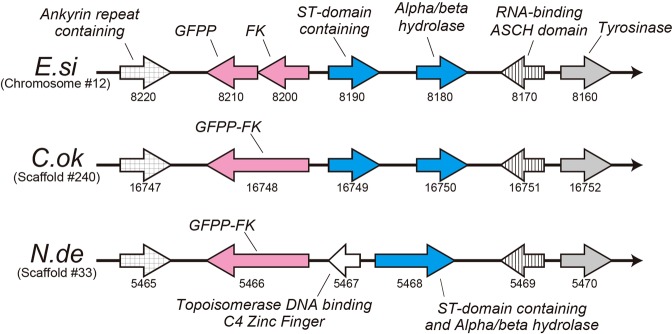
A diagrammatic representation of a syntenic region in genomes of three brown algae, *Ectocarpus siliculosus* (*E*.*si*), *Cladosiphon okamuranus* (*C*.*ok*) and *Nemacystus decipiens* (*N*.*de*). This region contains seven genes that encode Ankyrin repeat-containing protein, GDP-fucose pyrophosphorylase (GFPP), l-fucokinase (FK), ST-domain-containing protein, alpha/beta hydrolase, RNA-binding ASCH domain protein, and tyrosinase. In the *E*. *siliculosus* genome, all seven genes exist independently. However, in the *C*. *okamuranus* and *N*. *decipiens* genomes, the gene for l-fucokinase (FK) and the gene for GDP-fucose pyrophosphorylase (GFPP) are fused. In addition, in the *N*. *decipiens* genome, genes for ST-domain-containing protein and alpha/beta hydrolase are fused. These fusions are supported by corresponding mRNAs (Supplementary Figs S5 and S6), although the fused mRNA for the latter needs further examination. Insertion of a gene for topoisomerase DNA-binding C4 Zinc Finger protein was discovered in in the *N*. *decipiens* genome. Transcriptional direction is shown by the arrowhead. Numbers under genes indicate gene ID numbers.

Our previous study of the *C*. *okamuranus* genome found a possible fusion of the genes for l-fucokinase and GDP-fucose pyrophosphorylase (*FK-GFPP*)^14^, which was not found in the *E*. *siliculosus* genome (Figs 5 and 6). The present study confirmed that the genes are also fused in the *N*. *decipiens* genome (Fig. 6). There were no stop codons in the sequence of the transcript. The protein predicted by mRNA contained both the FK and GFPP domains (Supplementary Fig. S5). This suggests that the fused gene produces a bifunctional enzyme and that two enzyme-mediated processes are replaced by a single process. Although the function of the fused gene should be confirmed in the future, *N*. *decipiens* and *C*. *okamuranus* may have developed a more efficient means of producing sulfated fucans, compared to *E*. *siliculosus*.

The genomic region that contains *FK-GFPP* genes shows synteny among the three brown algae (Fig. 6). The *FK-GFPP* genes are inserted adjacent to an ankyrin repeat-containing gene at the 5′ flanking site and an ST-domain-containing gene, the alpha/beta hydrolase gene, the RNA-binding ASCH domain gene, and the tyrosinase gene on the 3′ flanking site. We found another possible fusion in the *N*. *decipiens* genome involving an ST-domain-containing gene with the alpha/beta hydrolase gene (Fig. 6 and Supplementary Fig. S5). Fusion seems probable because there were no stop codons in the sequences of the transcript and because RT-PCR analysis, in which two primers were designed to produce a ~2-kb single transcript resulted in a transcript of corresponding size (Supplementary Fig. S6). The ST-domain-containing gene was a component of 10 sulfotransferases. Although the function of the alpha/beta hydrolase has not been analyzed yet, this may be another means of facilitating sulfated fucan biosynthesis.

## Discussion

As described above, the present decoding of a draft genome of the “ito-mozuku” alga, *Nemacystus decipiens*, identified 15,156 protein-coding genes, approximately 78% of which were substantiated by corresponding mRNAs. CEGMA analysis showed that the *N*. *decipiens* genome assembly is of higher quality than those of the two other brown algae. To facilitate understanding of brown algal biology, we compared features of the three genomes. First, molecular phylogeny using 32 mitochondrial genes showed that *N*. *decipiens* and *C*. *okamuranus* share a more recent common ancestor. Although taxonomic classification of these brown algae should include morphological and life cycle data, the results appear to support the order Chordariales, including *N*. *decipiens* and *C*. *okamuranus*. An intimate relationship between *N*. *decipiens* and *C*. *okamuranus* can also be deduced from their morphology.

Our present analysis of genes for components of extracellular matrix (ECM) showed that 26 and 23 types of domain-containing genes are common in genomes of the brown algae and Chordariales, respectively. In contrast 16 domains were shared by Stramenopiles, and majority of domains was species specific (Fig. 4, Supplementary Fig S4, Supplementary Tables S7 and S8). This result was consistent with a previous report^21^, suggesting independent evolution of ECM-associated genes of the brown algae. The GlcNAc that is also annotated as glycosyl transferase family 60 was shared among *N*. *decipiens*, *C*. *okamuranus*, and *E*. *siliculosus*, whereas the glycosyl transferase family 2 gene was unique to *N*. *decipiens* and *C*. *okamuranus* (Supplementary Table S8). These results suggest that each organism has unique ECMs, whereas the glycosyl transferase family 60 gene is one of the key genes for polysaccharide biosynthesis in brown algae, and the glycosyl transferase family 2 was acquired and abundant in the Chordariales lineage.

A search for genes of enzymes involved in sulfated fucan biosynthesis identified all genes in this pathway. Our previous study demonstrated the fusion of genes for l-fucokinase (FK) and GDP-fucose pyrophosphorylase (GFPP), in the genome of *C*. *okamuranus*, but not *E*. *siliculosus*^14^. This suggests that “Okinawa mozuku” may have developed a more efficient way to synthesize sulfated fucans. The present study confirmed the presence of a fused gene of FK-GFPP in the *N*. *decipiens* genome as well. This fusion was supported by the corresponding mRNA. In addition, we found that the ST-domain-containing gene and the alpha/beta hydrolase gene are fused to each other in *N*. *decipiens* (Fig. 6). This fusion is evidenced by the lack of a stop codon between the sequences and by the results of RT-PCR analysis in which two primers designed to produce a ~2-kb transcript resulted in a single transcript of corresponding size (Supplementary Fig. S6). The ST-domain-containing gene was a sulfotransferase. Therefore, this draft genome of *Nemacystus decipiens* may provide a platform for future studies of sulfated fucan biosynthesis.

Cultivation of “ito-mozuku” in the Onna Fisheries Cooperative has a long history, commencing with the isolation of the “Ito5” strain in 1993 (Supplementary Fig. S1). We decoded the genome of the “Onna-1” strain, established in 2006. The Onna Fisheries Cooperative now maintains more than ten strains with different sporophyte morphology and responses to environmental changes. Due to world-wide environmental changes, including oceanic temperature rise, acidification, and pollution, brown algal culture is now facing critical conditions^11^. Continuous efforts toward maintenance and improvement are urgent. Genomic information about the “Onna-1” strain provides a reference for characterization of other strains with different features, and may facilitate subsequent improvement of “ito-mozuku” aquaculture to resist various environmental changes.

## Materials and Methods

### Biological materials

*Nemacystus decipiens*, “ito-mozuku” in Japanese, employed strains established and maintained by the Onna Fisheries Cooperative. The first, “Ito5,” was isolated from a wild population in 1993 (Supplementary Fig. S1A). The “Onna-1” strain was selected in 2006 and has been steadily maintained. This strain was used in the present study. It is cultivated at 22.5 °C with a 12-h light-dark cycle in sea water containing 0.5% KW21 (Daiichi Seimo Co. Ltd., Kumamoto, Japan).

The life cycle of *N*. *decipiens* includes both haploid (n) and diploid (2n) generations (Supplementary Fig. S1B)^4^. The 2n protonemas mature into sporophytes, and are harvested for market. Because the strain has been maintained as protonemas without contamination from other eukaryotes, it is easy to extract genomic DNA^14^, with protonemas as the dominant material.

### Frozen sections

Frozen sporophytes were embedded in Tissue-Tek O.C.T. compound (Sakura Finetek USA, Inc., Torrance, USA) and sectioned at 20 µm with Cryo-microtome CM3050S (Leica Microsystems GmbH, Wetzlar, Germany). Semi-thin sections were observed with an Axio Imager Z1 (Carl Zeiss, Oberkochen, Germany).

### DNA extraction, genome sequencing, and assembly

For DNA extraction, 2n protonemas of *N*. *decipiens* were frozen in liquid nitrogen and crushed to powder with a frozen-cell crusher, Cryo-Press (Microtec Co., Ltd, Chiba, Japan). Genomic DNA was extracted from the powder using a DNA-Suisui-VS extraction kit (Rizo Co., Ltd, Ibaraki, Japan). Illumina MiSeq and HiSeq 4000 platforms were used for sequencing^23^. Libraries were prepared with slight protocol modifications provided by the manufacturer. Fragmented genomic DNA was further purified using Blue Pippin (Sage Science, Beverly, MA, USA). A paired-end library consisting of 700-bp clones was prepared for the MiSeq using a TruSeq DNA PCR-Free LT Sample Prep Kit (Illumina, San Diego, CA, USA), and 2-, 3-, 4-, 5-, 6-, 7-, 9-, 11-, and 13-kbp mate-pair libraries were prepared for the HiSeq 4000 using a Nextera Mate Pair Sample Prep Kit (Illumina) (Supplementary Table S1). The BioProject ID was PRJDB7493.

K-mer counting and estimation of genome size were done with JELLYFISH 2.2.0 software^24,25^ and GenomeScope^26^. Adapter sequences were trimmed from all reads using Trimmomatic-0.30^27^. High-quality paired-end reads (quality >20) were assembled *de novo* using Platanus 1.2.4^28^ to create contigs. Subsequent scaffolding of the Platanus output was performed using SSPACE 3.0^29^, based on Illumina mate-pair information. Gaps inside scaffolds were closed using GapCloser 1.12^30^. Assembled sequences were aligned with blastn (1e^−50^) to another sequence. Sequences that aligned by more than 50% were removed as errors arising from diploid sequences. CEGMA 2.5 software^31^ was used to evaluate genome assembly. Sequences likely originated from bacteria and other microbiota were removed from the assembled genome with Maxbin version 2.2^32^ and RNAmmer 1.2^33^.

Paired-end genomic DNA reads that were not used in the *N*. *decipiens* genome were collected with kneaddata v0.6.1 (https://bitbucket.org/biobakery/kneaddata/wiki/Home). Those reads were assembled with novoPlasty (version2.7.2)^34^ for the chloroplast and mitochondrial genomes of *N*. *decipiens*.

### Transcriptome analyses

RNA was isolated from 2n protonemas (Supplementary Fig. S1B). Total RNA was extracted according to manufacturer instructions, using DNase and RNeasy Plant mini kits (QIAGEN, Hilden, Germany). Transcriptome libraries were prepared using a TruSeq Stranded mRNA Library Prep kit (Illumina). RNA was sequenced as per manufacturer instructions for the Illumina HiSeq 4000. Only sequences of high quality (quality >20) were assembled, using Velvet 1.2.10^35^ and Oases 0.2.08^36^.

### Gene model prediction

A set of gene model predictions (*Nemacystus decipiens* Gene Model ver. 1) was generated with AUGUSTUS 3.2.1^37^, which was trained on 9,793 transcriptome contigs recommended by PASA 2.2.0^38^. Gene models were produced by running AUGUSTUS on a repeat-masked genome, along with RepeatModeler-1.1.8 (http://www.repeatmasker.org/RepeatModeler.html), and refined with PASA.

### Transposable elements and repetitive sequences

Repetitive sequences were detected as described previously^39^. Tandem repeats were detected and classified using RepeatModeler. A *de novo* repeat library was generated with RepeatScout (version 1.0.5)^40^. Transposons and SINE in the scaffold were identified using RepeatMasker (ver. 4.0.7, http://www.repeatmasker.org/RMDownload.html) with the Repbase (version 21.01)^41^.

### Gene annotation and identification

In order to identify putative *N*. *decipiens* orthologous genes, reciprocal BLAST analysis was performed. This was carried out using mutual best hits of genes of *C*. *okamuranus*, *E*. *siliculosus*, and non-redundant protein sequences database from NCBI against *N*. *decipiens* gene models (BLASTP) or their assembly (TBLASTN). A second approach used for encoded proteins with one or more specific protein domains was to screen the models using HMMER (hmmer3)^42^ against the Pfam database (Pfam-A.hmm, release 24.0, http://pfam.sanger.ac.uk)^43^, which contains approximately 11,000 conserved domains. Encoded proteins were also analyzed using InterProScan 5.25–64.0^44^ for gene ontology annotations. The mitochondria genome was annotated with GeSeq^45^.

### Mitochondrial gene collection and Phylogenetic tree analysis

Sets of related sequences were subjected to phylogenetic analyses to more precisely determine orthologous relationships between *N*. *decipiens*, *C*. *okamuranus*, and *E*. *siliculosus*. Mitochondrial genomes sequences of 38 brown algae were downloaded from the NCBI database or our genome browsers (Supplementary Table S10). The mitochondrial genomes were annotated using GeSeq, and cDNA sequences of *Atp6*, *Atp8*, *Atp9*, *Cox1*, *Cox3*, *Cob*, *Nad1*, *Nad2*, *Nad3*, *Nad4*, *Nad4l*, *Nad5*, *Nad6*, *Nad7*, *Nad9*, *Rpl2*, *Rpl5*, *Rpl14*, *Rpl16*, *Rpl31*, *Rps2*, *Rps3*, *Rps4*, *Rps7*, *Rps8*, *Rps10*, *Rps11*, *Rps12*, *Rps13*, *Rps14*, *Rps19*, and *Tatc* genes from the 38 brown algae were collected. 32 gene sequences were independently aligned using MAFFT^46^ with default options. Spurious sequences or poorly aligned regions were filtered using trimAl^47^, then filtered sequences were concatenated. Phylogenetic trees were constructed by the maximum likelihood method (GTR-gamma model) using RAxML version 8.2.11^48^ with partition analysis excluded third codon and a 1,000 bootstrap replications.

### Searching extracellular matrix genes

Data of *N. decipiens, C. okamuranus, E. siliculosus, Thalassiosira pseudonana, Phytophthora infestans, Arabidopsis thaliana* and *Chlamydomonas reinhardtii* were downloaded from websites as shown in Supplementary Table S11. Downloaded protein sequences were first analyzed using signalP 4.1^49^, HECTAR^50^, and TMHMM 2.0^51^ to ensure that proteins contain signal sequences in their N-terminal, extra-membrane domains. Then, intracellular proteins were removed by searching for the endoplasmic reticulum targeting sequence (PDOC00014 in PROSITE database^52^) using MAST^53^. Collected proteins were defined as putative extracellular matrix proteins. Upset plots were drawn using UpSetR^54^.

### Identification of the orthologous gene group

Protein sequences of *N*. *decipiens*, *C*. *okamuranus*, and *E*. *siliculosus* were analyzed with OrthoFinder version 2.0.0^55^, using default parameters to identify orthologous gene groups.

### RT-PCR

cDNA was synthesized from total RNA with SuperScript™ IV First-Strand Synthesis System kit (Thermo Fisher Scientific Inc., Massachusetts, USA). Parts of coding regions of g5468 in the *N*. *decipiens* genome were amplified with PrimeSTAR GXL DNA Polymerase (Takara Bio Inc., Shiga, Japan). Primer sequences for the RT-PCR were 5′-TCTCCAAGACCGCCAAGG-3′ (Fw-primer) and 5′-TCAGCATCTTTCGCAGCC-3′ (Rv-primer). Blast analysis showed that these primers sequences were unique to the *N*. *decipiens* genome. PCR products were observed with an Agilent Bioanalyzer DNA 12000 kit (Agilent Technologies, California, USA) (Supplementary Fig. 5).

### Genome browser

A genome browser has been established for the assembled genome sequences using the JavaScript-based Genome Browser (JBrowse) 1.11.6^56^. The assembled sequence and gene models are accessible at http://marinegenomics.oist.jp/gallery/.

## Supplementary information

Supplementary Figures

Supplementary Tables

## References

[1] Van Den Hoek, C., Mann, D. G. & Jahns, H. M. *Algae: An Introduction to Phycology* (1995).

[2] Yoon HS, Hackett JD, Ciniglia C, Pinto G, Bhattacharya D (2004). A molecular timeline for the origin of photosynthetic eukaryotes. Mol. Biol. Evol..

[3] Silberfeld T, Rousseau F, Reviers Bd (2014). An Updated Classification of Brown Algae (Ochrophyta, Phaeophyceae). Cryptogamie, Algologie.

[4] Migita S, Yotsuji T (1972). Fundamental Studies on the Propagation of *Nemacystus decipiens*-I On the Life Cycle of *Nemacystus decipiens*. Bullet. Facul. Fisher..

[5] Yoshida T, Suzuki M, Yoshinaga K (2015). Checklist of Marine Algae of Japan (Revised in 2015). Jpn. J. Phycol. (Sôrui).

[6] Nisizawa K, Noda H, Kikuchi R, Watanabe T (1987). The Main Seaweed Foods in Japan. Hydrobiologia.

[7] Tako M, Nakada T, Hongou F (1999). Chemical Characterization of Fucoidan from Commercially Cultured *Nemacystus decipiens* (Itomozuku). Biosci. Biotechnol. Biochem..

[8] Baba M, Snoeck R, Pauwels R, De Clercq E (1988). Sulfated polysaccharides are potent and selective inhibitors of various enveloped viruses, including herpes simplex virus, cytomegalovirus, vesicular stomatitis virus, and human immunodeficiency virus. Antimicrob. Agents. Chemother..

[9] Lin TY, Hassid WZ (1966). Pathway of algnic acid synthesis in the marine brown alga, *Fucus gardneri Silva*. J. Biol. Chem..

[10] Michel G, Tonon T, Scornet D, Cock JM, Kloareg B (2010). The cell wall polysaccharide metabolism of the brown alga *Ectocarpus siliculosus*. Insights into the evolution of extracellular matrix polysaccharides in Eukaryotes. New Phytol..

[11] Porse H, Rudolph B (2017). The seaweed hydrocolloid industry: 2016 updates, requirements, and outlook. J. Appl. Phycol..

[12] Cock JM (2010). The *Ectocarpus* genome and the independent evolution of multicellularity in brown algae. Nature.

[13] Ye N (2015). *Saccharina* genomes provide novel insight into kelp biology. Nat. Commun..

[14] Nishitsuji K (2016). A draft genome of the brown alga, *Cladosiphon okamuranus*, S-strain: a platform for future studies of ‘mozuku’ biology. DNA Res..

[15] Yamada, N. Science of Seaweed Fucoidan. Seizando-shoten Publishing Co., Ltd. (2006).

[16] Cormier A (2017). Re-annotation, improved large-scale assembly and establishment of a catalogue of noncoding loci for the genome of the model brown alga *Ectocarpus*. New Phytol..

[17] Nishiyama T (2018). The *Chara* Genome: Secondary Complexity and Implications for Plant Terrestrialization. Cell.

[18] Gotz S (2011). B2G-FAR, a species-centered GO annotation repository. Bioinformatics.

[19] Jarvelainen H, Sainio A, Koulu M, Wight TN, Penttinen R (2009). Extracellular matrix molecules: potential targets in pharmacotherapy. Pharmacol. Rev..

[20] Daley WP, Peters SB, Larsen M (2008). Extracellular matrix dynamics in development and regenerative medicine. J. Cell. Sci..

[21] Terauchi M, Yamagishi T, Hanyuda T, Kawai H (2017). Genome-wide computational analysis of the secretome of brown algae (Phaeophyceae). Mar. Genomics.

[22] Saxena IMR, Malcolm Brown J, Fevre M, Geremia RA, Henrissat B (1995). Multidomain Architecture of b- Glycosyl Transferases: Implications for Mechanism of Action. Journal of Bacteriology.

[23] Bentley DR (2006). Whole-genome re-sequencing. Curr. Opin. Genet. Dev..

[24] Marcais G, Kingsford C (2011). A fast, lock-free approach for efficient parallel counting of occurrences of k-mers. Bioinformatics.

[25] Hirakawa H (2014). Dissection of the Octoploid Strawberry Genome by Deep Sequencing of the Genomes of Fragaria Species. DNA Res..

[26] Vurture GW (2017). GenomeScope: fast reference-free genome profiling from short reads. Bioinformatics.

[27] Bolger AM, Lohse M, Usadel B (2014). Trimmomatic: a flexible trimmer for Illumina sequence data. Bioinformatics.

[28] Kajitani R (2014). Efficient de novo assembly of highly heterozygous genomes from whole-genome shotgun short reads. Genome Res..

[29] Boetzer M, Henkel CV, Jansen HJ, Butler D, Pirovano W (2011). Scaffolding pre-assembled contigs using SSPACE. Bioinformatics.

[30] Li R (2010). The sequence and *de novo* assembly of the giant panda genome. Nature.

[31] Parra G, Bradnam K, Korf I (2007). CEGMA: a pipeline to accurately annotate core genes in eukaryotic genomes. Bioinformatics.

[32] Wu YW, Tang YH, Tringe SG, Simmons BA, Singer SW (2014). MaxBin: an automated binning method to recover individual genomes from metagenomes using an expectation-maximization algorithm. Microbiome.

[33] Lagesen K (2007). RNAmmer: consistent and rapid annotation of ribosomal RNA genes. Nucleic Acids Res..

[34] Dierckxsens N, Mardulyn P, Smits G (2017). NOVOPlasty: *de novo* assembly of organelle genomes from whole genome data. Nucleic Acids Res..

[35] Zerbino DR, Birney E (2008). Velvet: algorithms for de novo short read assembly using de Bruijn graphs. Genome Res..

[36] Schulz MH, Zerbino DR, Vingron M, Birney E (2012). Oases: robust *de novo* RNA-seq assembly across the dynamic range of expression levels. Bioinformatics.

[37] Stanke M, Diekhans M, Baertsch R, Haussler D (2008). Using native and syntenically mapped cDNA alignments to improve *de novo* gene finding. Bioinformatics.

[38] Haas BJ (2003). Improving the *Arabidopsis* genome annotation using maximal transcript alignment assemblies. Nucleic Acids Res..

[39] Takeuchi T (2012). Draft genome of the pearl oyster *Pinctada fucata*: a platform for understanding bivalve biology. DNA Res..

[40] Price AL, Jones NC, Pevzner PA (2005). De novo identification of repeat families in large genomes. Bioinformatics.

[41] Jurka J (2005). Repbase Update, a database of eukaryotic repetitive elements. Cytogenet. Genome Res..

[42] Eddy SR (1998). Profile hidden Markov models. Bioinformatics.

[43] Finn RD (2006). Pfam: clans, web tools and services. Nucleic Acids Res..

[44] Jones P (2014). InterProScan 5: genome-scale protein function classification. Bioinformatics.

[45] Tillich M (2017). GeSeq - versatile and accurate annotation of organelle genomes. Nucleic Acids Res..

[46] Katoh K, Misawa K, Kuma K, Miyata T (2002). MAFFT: a novel method for rapid multiple sequence alignment based on fast Fourier transform. Nucleic Acids Res.

[47] Capella-Gutierrez S, Silla-Martinez JM, Gabaldon T (2009). trimAl: a tool for automated alignment trimming in large-scale phylogenetic analyses. Bioinformatics.

[48] Stamatakis A (2014). RAxML version 8: a tool for phylogenetic analysis and post-analysis of large phylogenies. Bioinformatics.

[49] Petersen TN, Brunak S, von Heijne G, Nielsen H (2011). SignalP 4.0: discriminating signal peptides from transmembrane regions. Nat. Methods.

[50] Gschloessl B, Guermeur Y, Cock JM (2008). HECTAR: a method to predict subcellular targeting in heterokonts. BMC Bioinformatics.

[51] Krogh A, Larsson B, von Heijne G, Sonnhammer EL (2001). Predicting transmembrane protein topology with a hidden Markov model: application to complete genomes. J. Mol. Biol..

[52] Sigrist CJ (2013). New and continuing developments at PROSITE. Nucleic Acids Res..

[53] Bailey TL (2009). MEME SUITE: tools for motif discovery and searching. Nucleic Acids Res..

[54] Lex A, Gehlenborg N, Strobelt H, Vuillemot R, Pfister H (2014). UpSet: Visualization of Intersecting Sets. IEEE Trans. Vis. Comput. Graph..

[55] Emms DM, Kelly S (2015). OrthoFinder: solving fundamental biases in whole genome comparisons dramatically improves orthogroup inference accuracy. Genome Biol..

[56] Skinner ME, Uzilov AV, Stein LD, Mungall CJ, Holmes IH (2009). JBrowse: a next-generation genome browser. Genome Res..

