# Supplementary material for: Draft genome of the brown alga, Nemacystus decipiens, Onna-1 strain: Fusion of genes involved in the sulfated fucan biosynthesis pathway
Source: Sci Rep. 2019 Mar 14;9:4607. doi: 10.1038/s41598-019-40955-2 (PMC6418280; doi:10.1038/s41598-019-40955-2)
Supplement: Supplementary file 1 — Supplementary Figures [file 41598_2019_40955_MOESM1_ESM.pdf]

Draft genome of the brown alga, *Nemacystus decipiens*, Onna-1 strain: Fusion of genes involved in the sulfated fucan biosynthesis pathway

Koki Nishitsuji[1*], Asuka Arimoto[1], Yoshimi Higa[2], Munekazu Mekaru[2], Mayumi Kawamitsu[3], Noriyuki Satoh[1], and Eiichi Shoguchi[1]

[1]Marine Genomics Unit, Okinawa Institute of Science and Technology Graduate University, Onna, Okinawa 904-0495, Japan

[2]Onna Fisheries Cooperative, Onna, Okinawa 904-0414, Japan

[3]DNA Sequencing Section, Okinawa Institute of Science and Technology Graduate University, Onna, Okinawa 904-0495, Japan

----

[*] Correspondence should be addressed to: Tel. +81 98-966-8653. Fax. +81 98-966-2890. E-mail: koki.nishitsuji@oist.jp

Dr. Koki Nishitsuji, at Marine Genomics Unit, Okinawa Institute of Science and Technology Graduate University, Onna, Okinawa 904-0495, Japan
E-mail: koki.nishitsuji@oist.jp

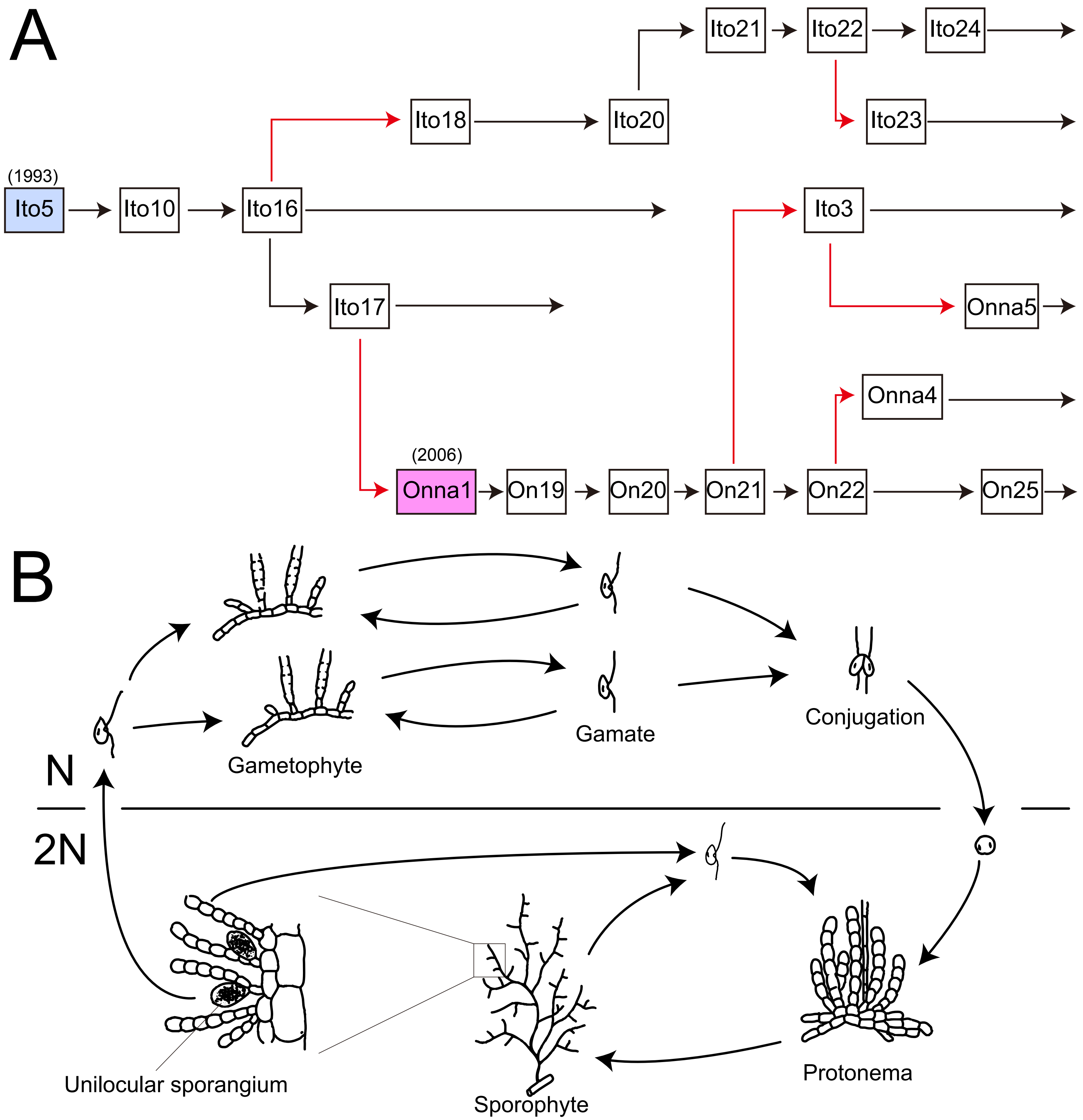

Supplementary Figure S1.

A. The lineage of strains of the *Nemacystus decipiens* that have been established and maintained by Onna Fisheries Cooperative. The "Ito5" (blue) was first isolated from the wild population in 1993. The "Ito17" strain was selected in 2006, and maintained to the present, and "Onna1" (magenta) was used in the present study. Black and red arrows indicate vegetative reproductions and sexual progenies.

B. The life cycle of *N. decipiens* has two generations of different nuclear phases (n and 2n). Genomic DNA and mRNA were extracted from protonemas.

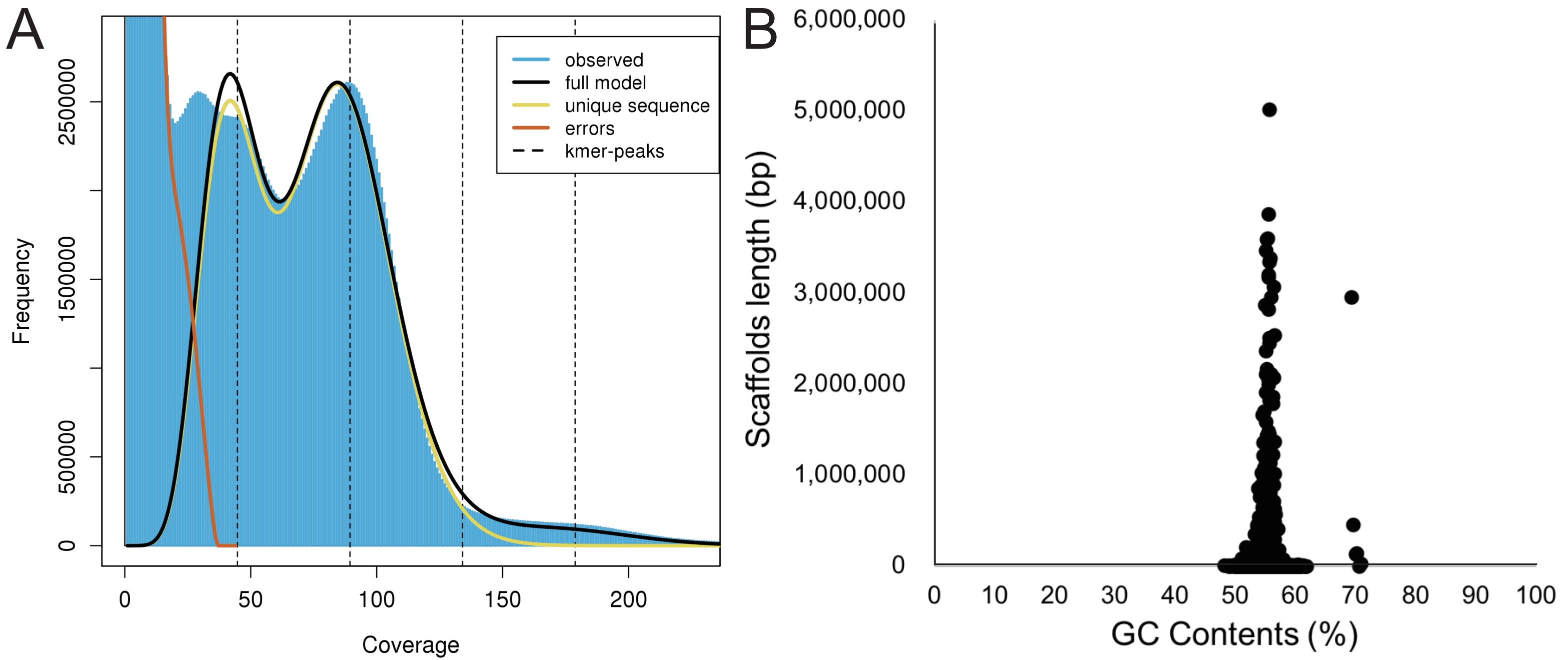

Supplementary Figure S2.

A. K-mer distribution at sequences of *Nemacystus decipiens* (K-mer = 32); the observed K-mer profile; full model, the fit of the GenomeScope model to the observed K-mer frequencies; unique sequence, heterozygous and homozygous sequences; errors, error rates; K-mer-peaks, observed K-mer peaks.

B. The plots of GC content of each scaffold comprising the *N. decipiens* genome.

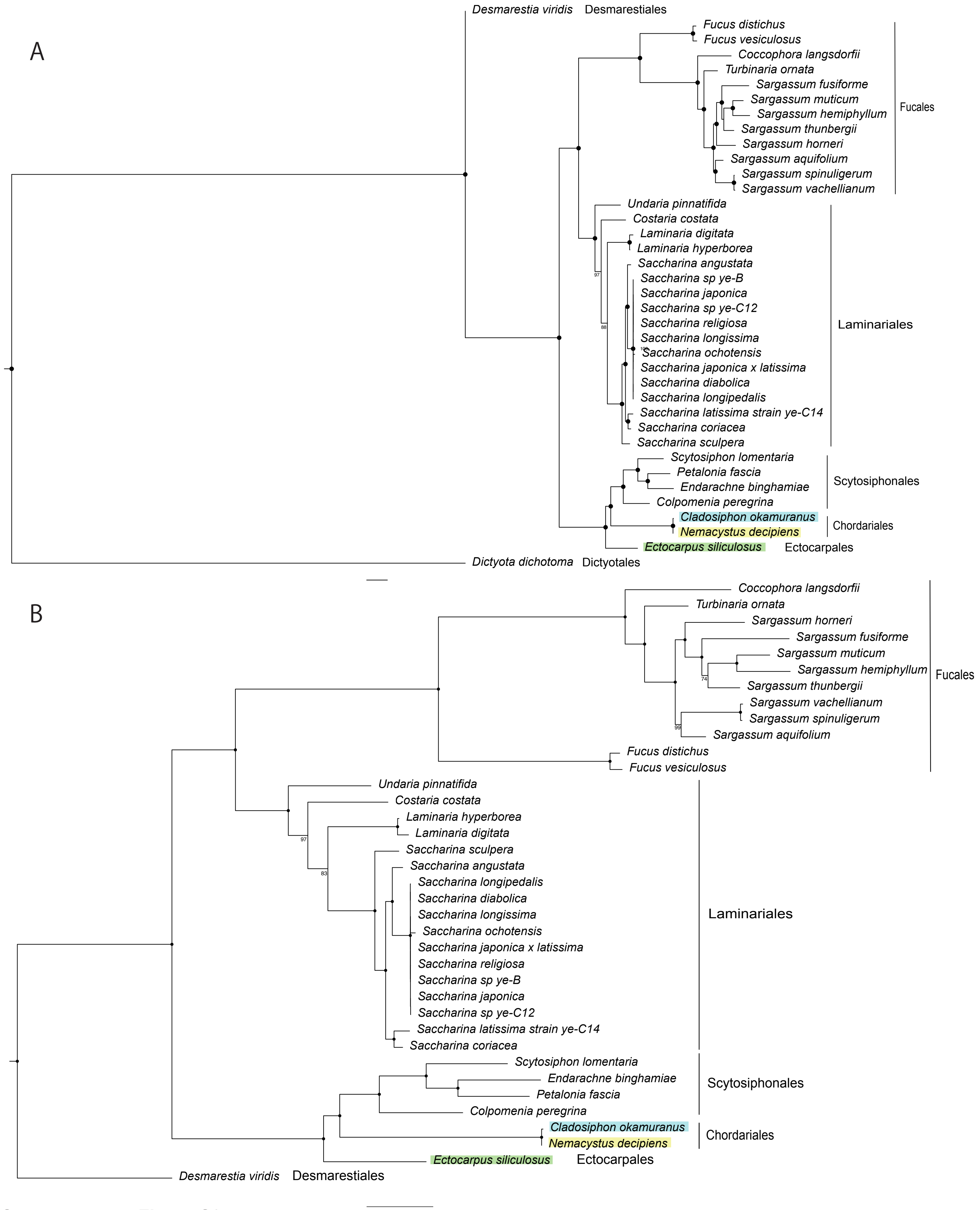

Supplementary Figure S3.

Phylogenetic trees of brown algae based on a comparison of 32 mitochondrial protein-coding gene sequences of 38 brown algae (A) and without *Dictyota dichotoma* (B).

The branch of *Desmarestia viridis* appeared to have disappeared was very long distance from *Dictyota dichoroma* (A). Re-analyzed phylogenetic tree without *D. dichoroma* was shown the branch of *D. viridis* (B).The phylogenetic relationship among other brown algae were conserved in between A and B. Black dots represent 100% bootstraps. Scale bars, 0.1 substitutions/site.

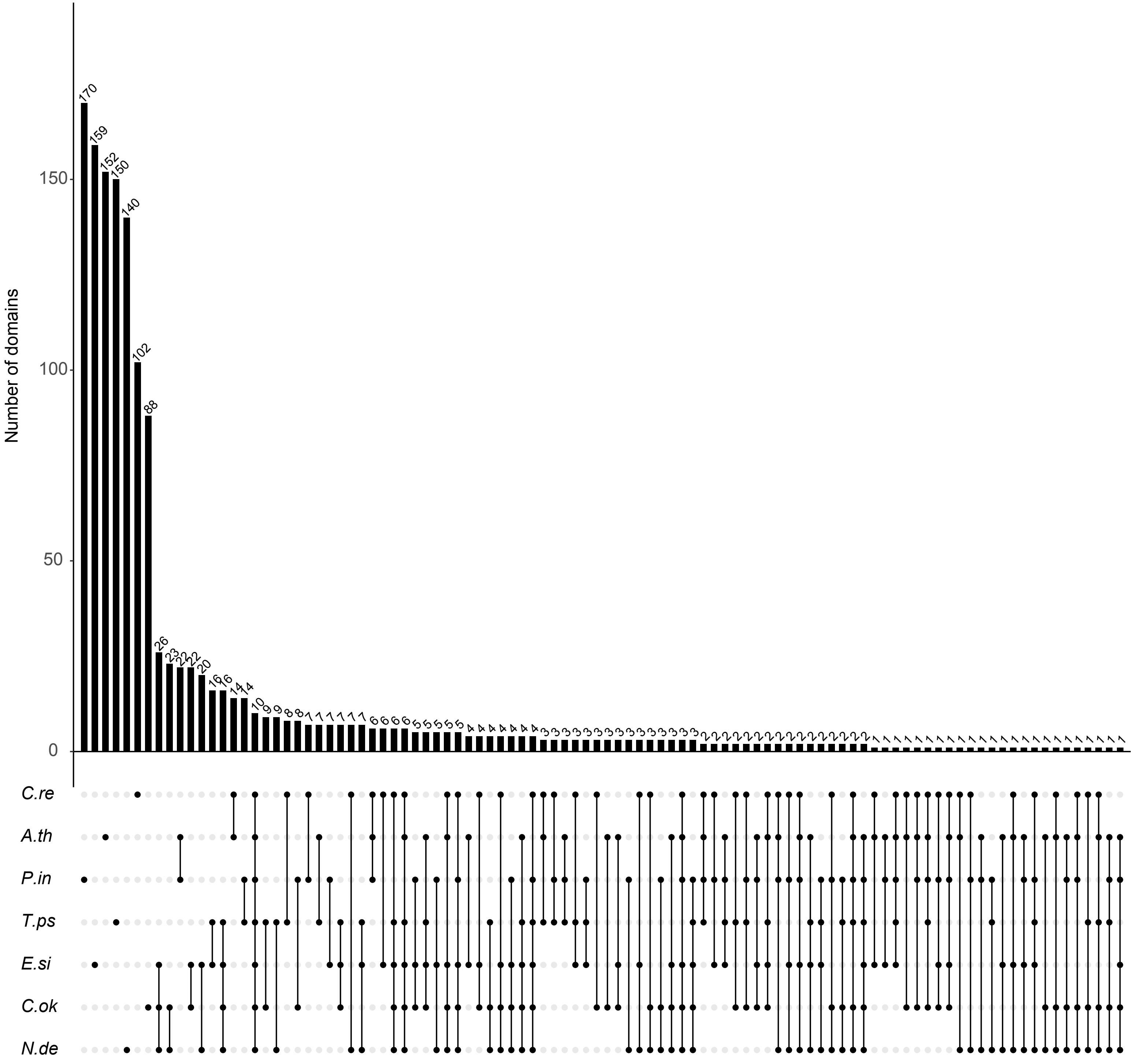

Supplementary Figure S4.

Upset plot associated to the extracellular matrix in the seven organisms. *N.de* indicates *Nemacystus decipiens*; *C.ok, Cladosiphon okamuranus*; *E.si, Ectocarpus siliculosus*; *T.ps, Thalassiosira pseudonana*; *P.in, Phytophthora infestans*; *A.th, Arabidopsis thaliana*; and *C.re, Chlamydomonas reinhardtii*. 10 domains were conserved among seven organisms, on the other hand, 3 domains were unique in Stramenopiles.

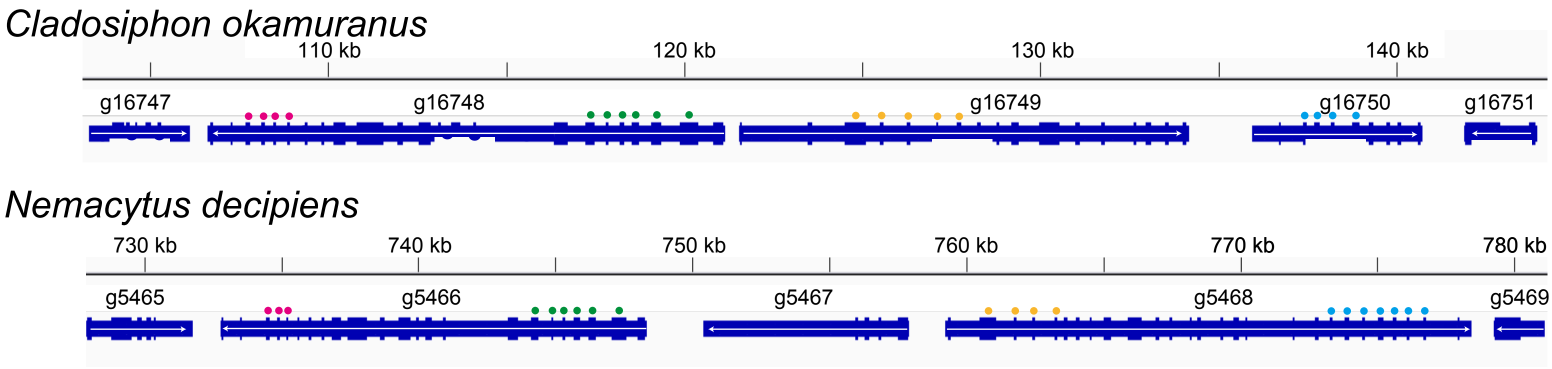

Supplementary Figure S5.

The genes which contain FK, GFPP, ST, and alpha/beta hydrolase domains in the *Cladosiphon okamuranus* and *Nemacystus decipiens* genomes.

The FK and GFPP domains are located in the g16748 gene in *C. okamuranus* genome and the g5466 gene in *N. decipiens* genome. The ST and alpha/beta hydrolase domains are located in the g16749 and g16750 genes in the *C. okamuranus* genome and the g5468 gene in *N. decipiens* genome. Colored dots indicate the domain containing exons. green dots; FK, magenta dots; GFPP, orange dots; ST, cyan dots; alpha/beta hydrolase.

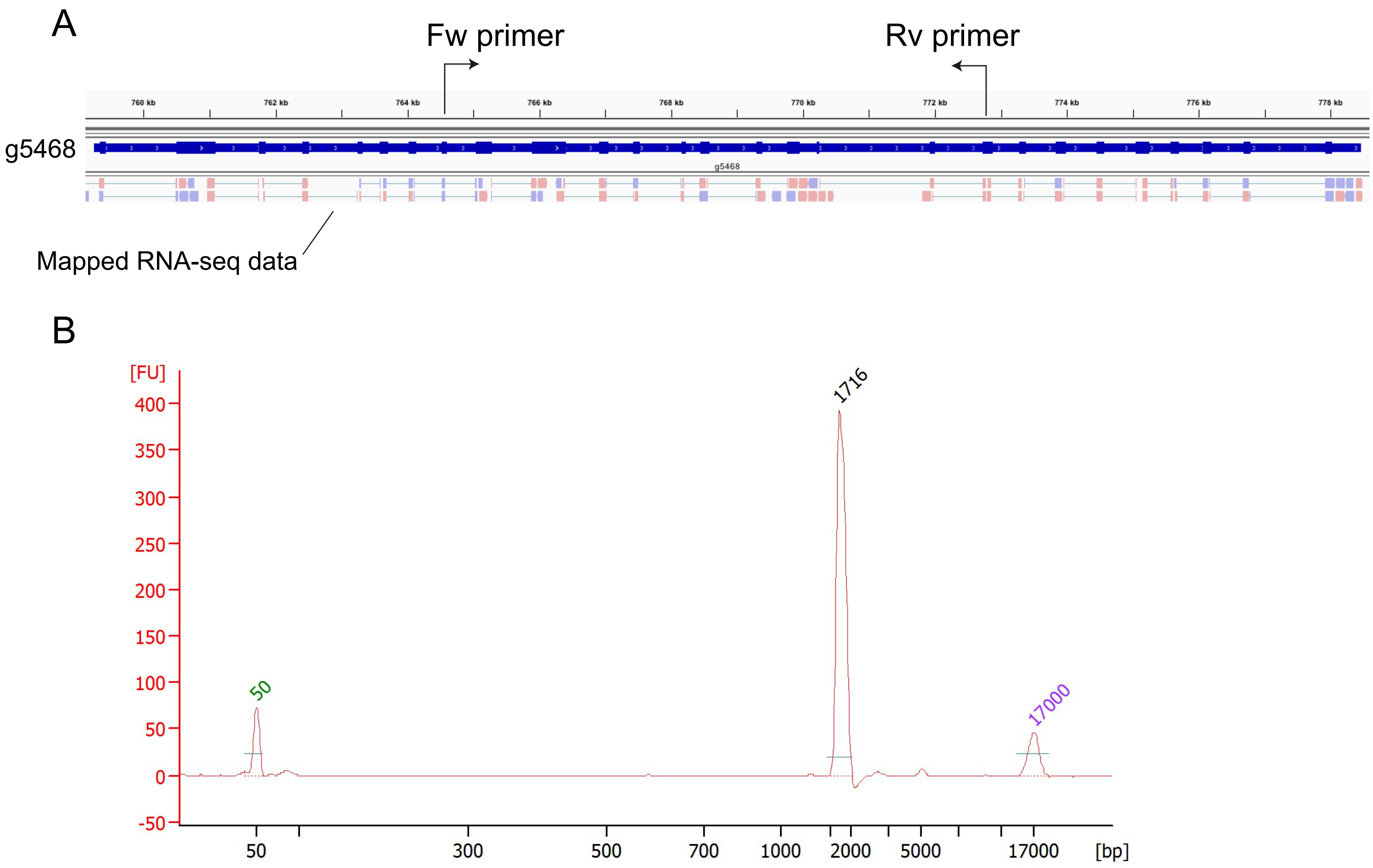

Supplementary Figure S6.

The primer sequences for the RT-PCR of g5468 in *Nemacystus decipiens* genome were 5’ -TCTCCAAGACCGCCAAGG-3’ (Fw-primer) and 5’ -TCAGCATCTTTCGCAGCC-3’ (Rv-primer) (A). The 1,716 bp DNA fragment was observed by Agilent bioanalyzer (B). Although there are variations of RNA-seq results, this result from RT-PCR supports the existence of g5468 gene model in *N. decipiens* genome.
